# Editorial: Update on Translational Neuroimmunology - Research of ISNI 2018

**DOI:** 10.3389/fimmu.2020.02012

**Published:** 2020-09-30

**Authors:** Lesley Probert, Francisco J. Quintana, Amit Bar-Or

**Affiliations:** ^1^Department of Immunology, Hellenic Pasteur Institute, Athens, Greece; ^2^Ann Romney Center for Neurologic Diseases, Brigham and Women's Hospital and Harvard Medical School, Boston, MA, United States; ^3^Center for Neuroinflammation and Experimental Therapeutics & Multiple Sclerosis Division, Department of Neurology, University of Pennsylvania, Philadelphia, PA, United States

**Keywords:** neuroimmunology, central nervous system, translational medicine, multiple sclerosis, neurodegeneration, immune system, biomarkers, immunotherapeutics

With its roots firmly planted in the fields of CNS autoimmunity and infection, the rapidly growing field of neuroimmunology is branching out to include almost every area of neurophysiology and neurological disease to encompass brain development and function, psychiatric illnesses, inflammatory demyelinating diseases, cancer, infections, and neurodegeneration of the central and peripheral nervous systems. The nervous system is also functionally intimately interconnected, via immune mechanisms, to multiple other processes and organ systems including systemic inflammation, gut microbiome, and chronic pain, to name a few. The scope of neuroimmunology and its implications for the understanding of human health and disease, from diagnosis through therapeutics, was highlighted during the 14th Congress of the International Society for Neuroimmunology (ISNI) 2018, and the associated 2nd Global Schools of Neuroimmunology (GSNI), held together in August 2018 in Brisbane, Australia. The richness of topics covered is further illustrated through the series of related articles presented in this special issue of Frontiers in Neurology entitled “Update on Translational Neuroimmunology Research of ISNI 2018.”

The Congress highlighted the importance taking the knowledge gained from translational research and clinical experience back into the research environment to gain further understanding of immune-mediated diseases affecting the human CNS and how to overcome them. During the meeting, dedicated symposia covered diverse topics of CNS and PNS autoimmunity, immune dysfunction, and immunotherapy, covering a wide range of neuroimmune interactions in health and disease. For example, disease topics ranged from immune-mediated conditions, such as multiple sclerosis (MS), neuromyelitis optica spectrum disorders (NMOSD), myasthenia gravis, and autoimmune encephalopathies and neuropathies, to inflammation in conditions usually thought of as neurodegenerative, such as Alzheimer's disease (AD), Parkinson's disease (PD), motor neuron diseases, neuropsychiatric diseases, as well as traumatic injuries and infections by parasites and neurotropic RNA viruses including West Nile and Zika.

The involvement of immune mechanisms in psychiatric diseases was introduced at GSNI on the first day of the meeting and during the main meeting in talks by Lennox and Brown. In this issue, McCombe et al. review the emerging evidence that the peripheral immune system contributes to motor neurodegeneration in amytrophic lateral sclerosis (ALS), and implications for therapeutic targeting to manage the disease. This parallels current thinking in AD that peripheral inflammation influences inflammation in the brain. In a similar vein, Yates et al. review the current evidence suggesting that extracellular vesicles (EVs) shed from CNS cells, cross the blood brain barrier (BBB), and play a critical role in activating peripheral immune responses to traumatic CNS injury. Vilquin et al. describe the consequences of autoimmune attack against components of the neuromuscular junction in myasthenia gravis, focusing on the response of the ultimate target in this disease, the muscle. Fujii et al. turn their attention to investigating the link between allergic inflammation and neuropathic pain, describing a possible role for spinal cord glial inflammation and autoantibodies that target sensory neurons in the dorsal root ganglia.

The dramatic clinical success of B cell depleting immunotherapies in patients with MS, as well as CNS diseases that involve autoantibodies such as NMOSD, has focused research on understanding the critical roles of B cells, and their interactions with T cells, in the pathogenesis of MS and other neuroimmune diseases. B cells produce antibodies, present antigens to T cells, and produce pro-inflammatory and anti-inflammatory cytokines, and all these functions can contribute to CNS autoimmunity. At the Congress, The Immunology Lecture delivered by Vinuesa was dedicated to rare mutations that contribute to systemic autoimmunity. Focus was placed on genes that control mechanisms involved in the production, selection and elimination of memory B cells and antibody production, and specifically on the role of roquin, an E3 ubiquitin ligase, in the control of B cell-T cell interactions by T follicular helper cells (TFH) (1). In the Dale McFarlin Lecture delivered by Martin, we learnt that memory B cells increase the spontaneous autoproliferation of peripheral T helper (Th1) cells as well as non-classical Th1 cells (CCR6+ CXCR3+; Th17.1 cells) (2). Importantly, T cell autoproliferation is abnormally increased in MS patients during the remission phase of the disease and is likely to drive disease (2). B cells do this in a HLA-DR1-dependent manner, and depletion of B cells by anti-CD20 antibodies reduces T-cell proliferation, thereby providing one mechanistic basis for therapeutic B cell depletion in MS. The same group identified a new putative target autoantigen in MS, RASGRP2, expressed in both brain and B cells (2). Insights from pediatric-onset MS discussed by Bar-Or also explored novel antigenic targets and emphasized the importance of cellular interactions between memory B cells, T cells and myeloid cells (3). Autoantibody-mediated pathologies of the peripheral and central nervous systems were a main topic of the meeting discussed by Vincent, De Seze, Mathey, Kusunoki and Kiernan. In this issue, van Langelaar et al. further discuss the types of T cell-B cell interactions that might be important for MS pathogenesis, Greer et al. report that autoantibodies to the major myelin protein, proteolipid 181–230 peptides, are increased in a subgroup of MS patients and that their levels positively correlate with disease severity, while Young reviews the differential effects of antibodies to the GluN1 subunit of the glutamate NMDA receptor in pathogenesis of neurological diseases.

Neutrophils are amongst the first immune cells to reach the CNS in the MS model experimental autoimmune encephalomyelitis (EAE) and are present in the CNS during NMOSD and severe MS, but little is known of their role in disease pathogenesis. Vallieres showed that neutrophils adopt macrophage-like properties once entering the CNS in a B cell-dependent EAE model and to promote inflammation via the neutrophil-specific protease ASPRV1 (4). Moreover, Korn discussed mechanisms by which polymorphonuclear myeloid-derived suppressor cells (PMN-MDSC), phenotypically related to neutrophils, control B cell accumulation and cytokine secretion during CNS autoimmunity (5). The involvement of other innate immune mechanisms in neurodegenerative disorders, such as heat shock proteins and the inflammasome, was also given attention by Amor, Issazadeh-Navikas, and Ting.

MS research is increasingly focused on myeloid cells and CNS-resident cells such as astrocytes, microglia, and oligodendrocytes, which contribute to disease pathology through additional mechanisms likely relevant for other neurologic diseases. One topic that remains hotly debated in the MS field is the identity of the antigen-presenting cell that is responsible for reactivating T cells in the CNS, and thereby licensing them to induce demyelinating lesions, since this is a potential target for more selective therapy. Various cell types, including dendritic cells, B cells, barrier-associated macrophages (BAM), and microglia are able to present antigen to T cells in the context of MHC class II, although which one is involved in the pathogenesis of EAE and MS still remains to be identified. Important targets are also the effector molecules communicating these interactions, as discussed by Becher for (GM-CSF) (6).

The functions of CNS-resident myeloid cell (microglia) in neuroinflammation continue to intrigue researchers. Unlike their peripheral counterparts (monocytes and macrophages), microglia perform essential functions in the CNS not only in pathologic states such as in repair of tissue damage, as discussed by Popovich, but also under normal physiologic conditions, such as shaping neuronal networks and clearing debris by phagocytosis. Loss of these functions during development has been implicated in schizophrenia and potentially in autism spectrum disorders, while loss of the same functions in the context of chronic inflammation may contribute to pathology in diseases such as MS and AD. For example, Waisman reported that selective depletion of A20 (an inhibitor of the transcription factor NF-κB) in microglia, was sufficient to induce spontaneous neuroinflammation and CNS infiltration by CD8+ T cells, pointing to a homeostatic function for microglia in the brain (7). The TAM family of receptor tyrosine kinases, Tyro3, Axl, and Mertk, has been implicated in demyelination, remyelination and MS susceptibility, and Kilpatrick reported that Mertk plays a role in beneficial microglia function in an experimental demyelination model, and that Tyro3 is important for developmental myelination of the CNS (8). In this issue, Benmamar-Badel et al. review data regarding a unique subset of microglia that is found in development, during normal aging and in diverse diseases and discuss the possible functional significance of these cells. It is clear that much more needs to be learnt regarding the differential functions of myeloid cell subtypes before these cells can be both effectively and safely targeted for therapy.

Evidence for inflammatory functions of CNS-resident oligodendrocyte precursor cells (OPC) was presented by Ransohoff wherein IL-17-induced Act1-NOTCH1 interactions in OPC promoted the inflammatory responses, cell proliferation and inhibited OPC differentiation into mature oligodendrocytes thereby inhibiting remyelination. Genetic depletion of NOTCH1 in OPC in mice, or administration of a decoy peptide based on IL-17RA, were sufficient to inhibit Th17-induced EAE (9). Increasing complexity of astrocyte function in disease is also being appreciated. Liddelow reported that astrocytes can be converted by microglia into a neurotoxic phenotype in diseases such as AD and PD, and agents that inhibit the formation of neurotoxic astrocytes could be used to treat these so-far untreatable diseases (10). Quintana described a novel metabolic mechanism involving cytosolic phospholipase A2 interactions with mitochondrial antiviral signaling protein (MAVS), which leads to activation of NF-κB and drives pro-inflammatory activities of astrocytes in EAE and MS, while interfering with the metabolic support of neurons by astrocytes. These findings identified a candidate drug to be repurposed for therapeutic modulation of astrocyte pro-inflammatory activities, while they also provide a novel link between viral infections, metabolism, CNS inflammation and neurodegeneration (11).

Our current understanding of CNS immune privilege and CNS barriers, including the blood brain barrier (BBB), blood CSF barrier (B-CSF), and brain surface barrier, was reviewed by Engelhardt at GSNI and elaborated during the congress itself, where the perivascular space at the BBB was implicated as a main entry point for immune cell infiltration in MS (12). Brain barrier dysfunction also occurs in other neurodegenerative diseases. Aging of the choroid plexus was described by Schwartz in an experimental AD model and was associated with the immune dysfunction and cognitive defects that are characteristic of this disease (13). Targeting of the choroid plexus using blockers of immune-inhibitory checkpoints, such as PD-1, evoked IFN-γ dependent immune responses, which in turn improved the recruitment of monocyte-derived macrophages including so-called disease-associated macrophages (DAMs), into the CNS and reduced disease features (13). These findings provide another example where the immune system exerts protective functions in the CNS during disease. Drugs that protect barrier function represent promising new therapies for MS, and as described by Yong include approved drugs such as minocycline, a tetracycline antibiotic used to treat acne, and novel inhibitor analogs of extracellular matrix components called chondroitin sulfate proteoglycans (CSPGs) (14).

Well-deserved attention was given to the gut-CNS axis and, in particular, the role of gut microbiota in immune homeostasis of the brain and in triggering CNS autoimmunity. This field developed following a landmark discovery made in the field of neuroimmunology just one decade ago. Since then the functional relationships between the gut microbiome and its effects on CNS pathology driven by peripheral and resident cells have been subject to intense investigation, as introduced at GSNI by Barazini, and updated in the Congress by Wekerle, Miyake, Weiner, Quintana and Yamamura, and by recent reviews (15–18). This special issue includes reviews from Jogia and Ruitenberg on the significance of gut microbiota in traumatic spinal cord injury, and from Cady et al., on the importance of dietary phytoestrogens in protection in EAE and possibly MS.

Several talks highlighted the neuroimmunology of infectious diseases of both the peripheral and central nervous systems, including cerebral malaria by Idro and viral infections by Klein, Pender, Basu and Yamano.

The search for improved diagnostic biomarkers continues, and is a particular focus of articles published in this special issue. While MS is the most common inflammatory demyelinating disease of the CNS, other rare disorders include NMOSD and anti-MOG associated disease, and the need for accurate diagnosis is stressed since prognosis and treatment of these three diseases are different. In this issue, Prain et al. describe a clinically-based survey of NMOSD in Australia and New Zealand, and critically evaluate different assays for anti-AQP4 and anti-MOG antibodies used in the diagnosis of NMOSD and anti-MOG associated disease, respectively. In the case of anti-AQP4 antibody seronegative NMOSD, differential diagnosis from MS might be difficult. The current status of medical imaging research in MS and NMOSD, and its role in the diagnosis and management of these two diseases was discussed at the meeting by Stankoff and Paul, and is reviewed in this issue by Kuchling and Paul. Tea et al. together with the Australasian and New Zealand MOG Study Group, discuss data analysis tools for maximizing the diagnostic power of FACs cell-based assays that detect MOG autoantibodies. Gastaldi et al. critically report the results and main shortcomings of the 2018 Italian Neuroimmunology Association external quality assessment program (EQAP), which evaluated assays using a wide range of markers including oligoclonal bands, antibodies to intracellular and surface neuronal antigens, AQP4, MOG, and myelin-associated glycoprotein (MAG) antibodies, in different assay types used by 34 different laboratories. Jiang et al., describe improved novel biomarkers in the CSF of patients with autoimmune encephalitis for differential diagnosis between viral infections and autoimmune encephalitis. Masvekar et al. investigated whether apoptotic bodies/ apoptosomes, which are vesicles released from apoptotic cells, could represent a biomarker for MS, by measuring total and cell-specific apoptotic bodies in the CSF of MS patients by FACS using annexin V and antibodies to cell-specific markers.

Novel immunotherapeutic strategies for wide range of peripheral and central nervous system diseases, including neuropathies, myasthenia gravis, and pediatric CNS disorders were discussed during the meeting. The powerful therapeutic value of neural stem cells was the subject of The John Newsom David Lecture delivered by Martino (Ottoboni et al.) and The Rita Levi-Montalcini Neurobiology Lecture delivered by Bartlett (19), and is further highlighted in this special issue in a review by Ottoboni et al.. News of a phase I safety trial using allogenic human NSC in MS patients with progressive MS was shared by Martino, and strategies for further improvement of this approach by using autologous induced pluripotent stem cell derived NSC were considered. The homeostatic functions of NSC in brain inflammation and their contribution to remyelination and brain repair in experimental models was reported. Under conditions of inflammation, NSC are maintained in an immature state and secrete LIF, which in turn promotes remyelination. In a separate report by Kilpatrick, oligodendrocyte-specific expression of the TAM receptor Tyro3 was necessary for remyelination in a demyelination model in mice (8). The possibility that these mechanisms interact to mediate CNS repair in demyelinating disease remains to be investigated. On the other hand, positive regulators of neurogenesis from NSC in the hippocampus are found to be stimulated by exercise and healthy lifestyle (19). The importance of microglial and astrocytic gap junction proteins in the modulation of CNS pathology was pointed out in talks by Suzumura and Kira. In this issue, Finardi et al., provide evidence that microRNAs miR106b-25 and miR17-92, which are upregulated in MS patient T regulatory cells, are involved in the development of experimental neuroinflammation and might represent novel therapeutic targets. In the context of glioma, in this issue Sarkar et al. provide evidence that the antibiotic demeclocycline reduces the growth of brain tumor initiating cells through direct and indirect effects via activation of myeloid cells.

Further advancing the spirit of translational research in the neuroimmunology field, the importance of improved animal models of autoimmunity and infection was highlighted in the talks by Liblau, Baker, De Seze, Klein and O'Connor. Several new animal models have been developed to recapitulate findings from the clinic and to aid deeper mechanistic studies into disease pathogenesis. A humanized mouse model for Rasmussen's encephalitis was generated by transplanting patient PBMC's into immunodeficient NSG mice, as reported by Prat (20), as were B cell models for MS and NMOSD, including one in which sequences encoding human AQP 4 antibody isolated from an NMOSD patient were knocked into the mouse heavy chain locus as reported by Kuchroo (21), and another EAE model induced by immunization with AQP4 201-220 peptide in AQP4 KO mice as reported by Zamvil (22). In this issue, Giannoccaro et al. review results and the limitations of current animal models of autoantibody-mediated neurological diseases, and discuss the increasing evidence that maternal antibodies to neuronal surface antigens in the maternal circulation can reach the fetal brain during gestation causing neurodevelopmental disorders.

In summary, this collection represents the broad range of NeuroImmunology research presented at the 14th Congress of the International Society for Neuroimmunology (ISNI) 2018, and the associated 2nd Global Schools of Neuroimmunology (GSNI), highlighting new developments in this rapidly moving field, as well as unanswered research questions and unmet clinical needs. This growing body of research sets up the stage for the upcoming ISNI/GSNI 2021 meeting in Nice, France.

## Author Contributions

All authors listed have made a substantial, direct and intellectual contribution to the work, and approved it for publication.

## Conflict of Interest

The authors declare that the research was conducted in the absence of any commercial or financial relationships that could be construed as a potential conflict of interest.
